# Circumferential Negative Pressure Lymphedema Therapy: A Novel Rapid Perioperative Decongestion Strategy for Advanced Stage III-IV Lymphedema

**DOI:** 10.7759/cureus.103526

**Published:** 2026-02-13

**Authors:** Sumita Shankar, Mallavarapu Chandralekha, Chimata Nikhila, Kruthi Nanduri, S Vijaya, D Navya Sesha Harika

**Affiliations:** 1 Department of Plastic and Reconstructive Surgery, Guntur Medical College, Guntur, IND

**Keywords:** decongestive, lymphatic drainage, lymphatic filariasis, lymphedema, negative pressure

## Abstract

Introduction: Advanced Stage III-IV lymphedema, particularly in filariasis-endemic regions, presents with severe fibrosis, massive limb enlargement, and a poor response to conventional conservative therapy, complicating reductive surgical interventions in resource-limited settings. This study evaluated circumferential negative pressure lymphedema therapy (CNPLT), a novel rapid preoperative decongestion technique, for transforming inoperable limbs into suitable surgical candidates.

Methods: This prospective clinical study enrolled 14 patients (16 limbs) with Stage III-IV lower extremity lymphedema at a high-volume public tertiary hospital in India (September 2022-March 2024). Patients underwent intensive CNPLT (continuous subatmospheric pressure 200-250 mmHg via circumferential polyurethane foam dressings) for seven to eight days preoperatively. Limb circumference was measured daily at four standardized levels (mid-foot, lateral malleolus, 10 cm, and 20 cm above the malleolus). Body weight, 24-hour urine output, and skin suppleness were monitored. Following clinical readiness, patients underwent lympho-liposuction or excisional reductive surgery. Statistical analysis was performed using paired t-tests, repeated-measures analysis of variance, post hoc analysis with the Bonferroni test, and McNemar’s test (p < 0.05).

Results: CNPLT produced highly significant limb circumference reductions at all sites: mid-foot (5.7 ± 1.2 cm), lateral malleolus (17.5 ± 2.8 cm), 10 cm above (17.4 ± 3.1 cm), and 20 cm above (14.0 ± 2.8 cm) (all p = 0.001). Mean body weight decreased from 91.5 ± 12.6 to 72.9 ± 15.8 kg presurgery (p = 0.001), with urine output rising from 1,200 ± 250 to 1,850 ± 300 mL/day (p = 0.001). Skin laxity improved markedly, with complete resolution increasing from 14.3% preoperatively to 85.7% preoperatively (p = 0.001). Reductive surgery was performed safely; minor wound dehiscence occurred in six cases and resolved conservatively. No major complications (necrosis, infection, or seroma requiring intervention) were observed. Post hoc analyses with Bonferroni correction revealed significant reductions in both weight and urine output from the pre‑CNPLT stage to the presurgery and postsurgery stages (all p < 0.001). The mean hospital stay was 12-13 days.

Conclusion: CNPLT offers a safe, rapid, and effective preoperative decongestive strategy for advanced lymphedema, achieving substantial volume reduction, systemic fluid mobilization, and tissue softening within one week. This technique facilitates safer surgery, shortens hospitalization, and has strong potential in high-burden endemic settings.

## Introduction

Lymphedema is a chronic progressive disorder resulting from impaired lymphatic drainage, which leads to the accumulation of protein-rich interstitial fluid and subsequent tissue fibrosis, adipose deposition, and massive limb enlargement [1]. In tropical countries such as India, lymphatic filariasis remains the predominant cause of advanced (Stage III-IV) lymphedema, characterized by severe induration, nonpinchable skin, deep folds, recurrent cellulitis, and irreversible structural remodeling that renders the limb rigid and functionally disabling [2]. Secondary lymphedema following cancer treatment and its rare congenital forms also contribute to this challenging clinical spectrum.

In advanced stages, conventional conservative management, primarily complete decongestive therapy (CDT) involving manual lymphatic drainage (MLD), multilayer compression bandaging, and meticulous skin care, often yields only modest or delayed improvement, typically requiring weeks to months of strict adherence [3,4]. In high-volume public tertiary hospitals, where surgical resources are heavily committed to acute trauma and emergencies, prolonged preoperative preparation is often impractical. Moreover, attempting reductive surgery (lympho-liposuction or excisional procedures) on heavily fibrosed, noncompliant limbs significantly increases the risk of postoperative complications, including wound dehiscence, skin flap necrosis, seroma formation, and delayed healing [5].

Therefore, there is an urgent need for innovative, rapid preoperative decongestive strategies that can physiologically mobilize large volumes of interstitial fluid, soften the fibroadipose envelope, and transform “inoperable” limbs into safe, supple surgical targets within a clinically feasible timeframe. Circumferential negative pressure lymphedema therapy (CNPLT) addresses this gap by circumferentially applying continuous subatmospheric pressure (200-250 mmHg) through polyurethane foam dressings, creating a mechanical macrostrain that distends the dermis, engages lymphatic anchor filaments, opens initial lymphatic endothelial junctions, and promotes systemic diuresis of mobilized fluid. This prospective study aimed to evaluate the efficacy and physiological impact of CNPLT as a rapid perioperative decongestion strategy in patients with advanced Stage III-IV lymphedema. The specific objectives were to quantify limb circumference reduction at standardized anatomical levels; document systemic fluid mobilization through body weight changes and daily urine output; assess improvement in skin suppleness and laxity to facilitate safer reductive surgery; and observe postoperative outcomes, hospital stay duration, and early complications in a high-volume public hospital setting.

## Materials and methods

Study design and setting

This prospective clinical study was conducted in the Department of Plastic and Reconstructive Surgery of the Guntur Medical College, Guntur, Andhra Pradesh, India, from April 2023 to December 2024. Institutional ethical approval was obtained (approval no.: GMC/IEC/027/2023), and all participants provided written informed consent for the CNPLT procedure, surgery, photography, and publication.

Patient selection and demographics

Because CNPLT is a novel technique with no prior published data on effect size or variability, a formal power-based sample size calculation was not performed. The study followed the exploration phase of the ideal framework for surgical innovation, in which small prospective case series (10-30 patients) are the standard for establishing feasibility, safety, and preliminary efficacy.

**Figure 1:**
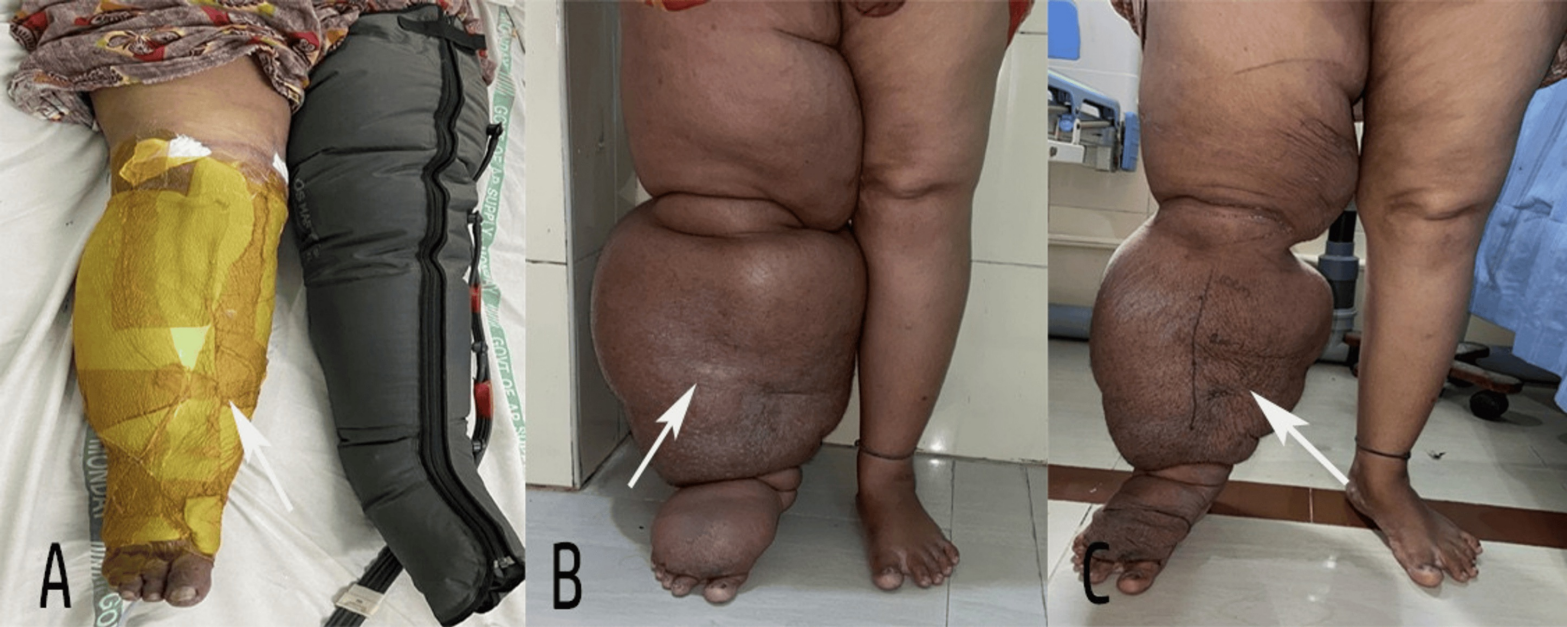
(A) Preoperative clinical presentation with bilateral lymphedema CNPLT applied on the right leg and conventional pneumatic compression device on the left leg. (B) Preoperative clinical presentation with unilateral lymphedema. (C) Post-CNPLT view showing improvement on the affected side CNPLT: circumferential negative pressure lymphedema therapy

Fourteen patients (16 limbs) with advanced stage III-IV lymphedema were enrolled [6]. The inclusion criteria were Grade III or IV lymphedema (limb enlargement >30% compared to the contralateral side, severe fibrosis, lobulations, deep skin folds, or ulceration) and candidacy for definitive reductive surgery (lympho-liposuction or excisional procedures) (Figure 1A). The etiologies included endemic filariasis, congenital lymphatic malformations, and secondary causes. Exclusion criteria were coexisting venous or arterial insufficiency, deep vein thrombosis, active systemic infection or sepsis, or inability to comply with perioperative compression. One patient with hypertension was stabilized preoperatively and included. The cohort consisted of nine male and five female patients (mean age 42.6 years, range 20-65 years), with 11 right-sided, five left-sided, and two bilateral cases. One patient had a chronic nonhealing ulcer of four years’ duration. Patients were excluded if they had known congestive heart failure, reduced left ventricular ejection fraction (160/100 mmHg despite treatment), recent myocardial infarction, or severe valvular disease. One patient with controlled hypertension (on monotherapy) was included after cardiologist clearance and preoperative optimization.

Preoperative CNPLT protocol

Upon admission, the affected limb was thoroughly cleaned using an antiseptic solution. Medical-grade open-cell polyurethane foam (1 cm thick, pore size 0.2-1 mm) was applied circumferentially, extending from the distal foot to the proximal limit of edematous tissue (typically the mid-thigh or groin), conforming to skin folds and irregularities (Figure 1B). A fenestrated evacuation tube was embedded within the foam and connected to a continuous negative-pressure source. The therapeutic pressure was set at 200-250 mmHg; in cases of fragile skin or ulceration, it was reduced to 160 mmHg for safety. The dressing was secured using semipermeable occlusive adhesive drapes to ensure an airtight seal. CNPLT was applied intensively for seven to eight days in the "Metabolic Monitoring Phase," with two sessions before surgery. The patients were then hospitalized for close observation. Daily clinical monitoring included assessment for signs of intravascular overload (dyspnea, orthopnea, crackles, and elevated jugular venous pressure). Serum electrolytes, renal function tests, and hemoglobin were checked on days 1, 4, and 7 (or presurgery). No supplemental intravenous fluids were administered unless clinically indicated; oral fluid intake was unrestricted but encouraged to match enhanced diuresis.

Measurements and monitoring

The primary outcomes were rapid decongestion and systemic fluid mobilization (Figure 1C). Limb circumference was measured daily at four standardized anatomical landmarks: mid-foot, lateral malleolus, 10 cm above the malleolus, and 20 cm above the malleolus using a nonstretch tape measure. Limb circumference was measured daily with the vacuum-assisted closure therapy temporarily paused, and the outer occlusive drape gently opened, while the underlying polyurethane foam was left in situ to prevent tissue recoil. Measurements were taken over the compressed foam at fixed anatomical landmarks using a nonstretch tape by the same observer each day, ensuring consistent serial assessment. The dressing was immediately resealed, and negative pressure therapy resumed at the prescribed settings. Body weight was recorded daily, and 24-hour urine output was monitored as an indicator of interstitial fluid shift into systemic circulation and subsequent diuresis. Skin texture and laxity were assessed subjectively and clinically (transition from nonpinchable/indurated to supple/pinchable skin). Vital signs and metabolic stability were tracked to ensure safety during the initial fluid surges.

Surgical protocol

**Figure 2:**
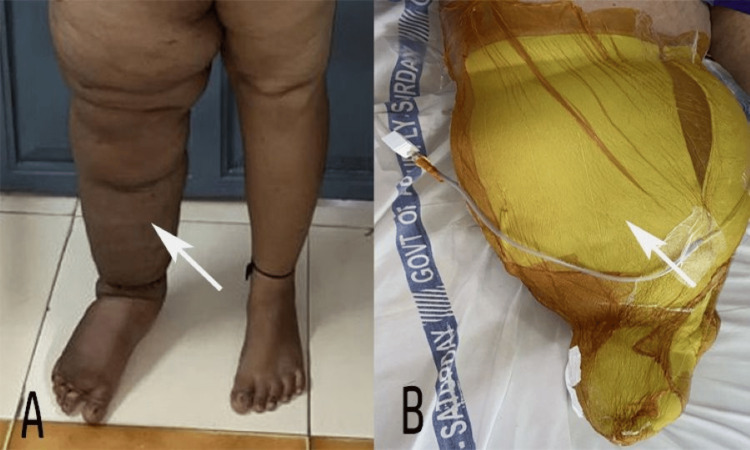
(A) Postoperative improvement in the right leg. (B) CNPLT in place covering the limb uniformly CNPLT: circumferential negative pressure lymphedema therapy

Following the achievement of clinical "surgery-readiness" (marked softening, significant girth reduction, and pinchable skin), patients underwent definitive reductive surgery under spinal anesthesia. Lympho-liposuction was performed using a 3-mm cannula under tourniquet control without tumescent infiltration due to advanced fibrosis. Excisional removal of redundant fibrotic tissue ensured tension-free closure, and flap elevation was avoided. The bilateral cases were addressed in a single session. One patient required an intraoperative transfusion. Immediately after closure, CNPLT was reapplied for four days, supplemented with suction drains (Figures 2A, 2B). The suture line was inspected during routine daily dressing assessments by temporarily pausing negative-pressure therapy and gently opening the occlusive drape over the incision without disturbing the underlying foam. The wound edges were assessed clinically for color, tension, discharge, and early dehiscence, after which the drape was resealed, and negative-pressure therapy was promptly resumed.

Postoperative care and follow-up

Compression stockings were fitted after removal of the postclosure CNPLT, once the negative pressure dressing was discontinued and the surgical wounds were inspected to confirm satisfactory hemostasis and incision integrity. The patients were discharged on postoperative day 5 and were reviewed on day 10. Minor wound dehiscence (observed in six cases) was managed conservatively without secondary intervention. Follow-up was continued for up to six months to assess sustained volume reduction, wound healing, absence of recurrence (ulceration or lymphorrhea), and overall complications (seroma and infection). The duration of hospital stay was recorded.

Statistical analysis

Statistical analyses were performed using the Statistical Package for Social Sciences software (version 23.0, IBM Corporation, Armonk, NY). Data are presented as mean ± standard deviation or frequency (percentage). The primary analysis compared the pre-CNPLT and post-CNPLT (presurgery) time points. Normality was assessed and confirmed using the Shapiro-Wilk test. For continuous outcomes (limb circumference, weight, and urine output), paired t-tests were used. For categorical data (skin laxity), the McNemar's test was used. Secondary analysis across all three time points employed a repeated-measures analysis of variance test, followed by post hoc analysis. Statistical significance was set at p <0.05.

## Results

No patient developed clinical or radiological evidence of pulmonary edema, acute heart failure, or symptomatic electrolyte imbalance during CNPLT. Minor asymptomatic hyponatremia (Na 132-134 mmol/L) occurred in two cases and resolved spontaneously with oral fluid adjustment. The study cohort comprised 14 patients, with a mean age of 42.6 ± 18.43 years and predominantly nine (64.3%) male patients. The mean baseline weight was 91.5 ± 12.6 kg. This indicates a middle-aged, predominantly male sample presenting with obesity, which is a common profile for severe lower extremity lymphedema (Table 1).

**Table 1:** Demographic profile of patients with advanced Stage III-IV lymphedema treated with circumferential negative pressure lymphedema therapy followed by reductive surgery (n = 14) Continuous data (age and weight) are presented as mean and SD, and categorical data (sex) are presented as frequency (n) and percentage (%) SD: standard deviation

Parameters	Value
Age (years), mean ± SD	42.6 ± 18.43
Sex, n (%)	
Male	9 (64.3%)
Female	5 (35.7%)
Baseline weight (kg), mean ± SD	91.5 ± 12.6

The results demonstrated that CNPLT produced statistically significant reductions in limb circumference at all measured anatomical sites (p = 0.001). Substantial and consistent decreases were observed, with the greatest absolute reduction at the lateral malleolus (17.5 ± 2.8 cm) and 10 cm above it (17.4 ± 3.1 cm). Significant reductions were also noted at the mid-foot (5.7 ± 1.2 cm) and 20 cm above the malleolus (14.0 ± 2.8 cm). Uniformly high t-values confirm a robust treatment effect. These data suggest that CNPLT is a highly effective decongestive intervention for lower limb lymphedema, successfully reducing excess volume and tissue edema throughout the distal leg and ankle region prior to definitive surgical management (Table 2).

**Table 2:** Circumferential limb measurements before and after seven to eight days of CNPLT ^*^Statistically significant (p < 0.05) using paired t-test Data are presented as mean and SD CNPLT: circumferential negative pressure lymphedema therapy; SD: standard deviation

Anatomical site	Pre-CNPLT (cm), mean ± SD	Presurgery (cm), mean ± SD	Mean reduction (cm), mean ± SD	t value	p value
Mid-foot	36.2 ± 4.2	30.5 ± 3.1	5.7 ± 1.2	17.77	0.001^*^
Lateral malleolus	65.7 ± 5.6	48.2 ± 3.2	17.5 ± 2.8	23.39	0.001^*^
10 cm above the lateral malleolus	68.6 ± 4.8	51.3 ± 5.4	17.4 ± 3.1	21.00	0.001^*^
20 cm above the lateral malleolus	60.7 ± 3.8	46.6 ± 4.9	14.0 ± 2.8	18.71	0.001^*^

These results confirmed that CNPLT induced significant physiological and clinical changes prior to surgery. Patient weight decreased substantially from 91.5 ± 12.6 to 72.9 ± 15.8 kg (p = 0.001), with a further reduction postoperatively, indicating effective fluid and tissue decongestion. Concurrently, urine output increased significantly from 1,200 ± 250 to 1,850 ± 300 mL/day (p = 0.001), reflecting enhanced renal fluid mobilization. Skin laxity also improved markedly, with the rate of complete resolution increasing from 14.3% to 85.7% preoperatively (p = 0.001). Collectively, these findings suggest that CNPLT is a potent decongestive therapy that effectively reduces limb volume, augments fluid excretion, and improves skin quality, thereby optimizing patient conditions for subsequent surgical intervention (Table 3).

**Table 3:** Changes in systemic parameters and skin laxity before CNPLT, after CNPLT (presurgery), and after surgery ^†^Repeated-measures analysis of variance f value ^‡^McNemar's test statistic ^*^Statistically significant (p < 0.05) CNPLT: circumferential negative pressure lymphedema therapy; SD: standard deviation

Parameters	Pre-CNPLT	Presurgery	Postsurgery	Test value	p value
Weight (kg), mean ± SD	91.5 ± 12.6	72.9 ± 15.8	69.0 ± 12.5	25.73^†^	0.001^*^
Urine output (mL/day), mean ± SD	1,200 ± 250	1,850 ± 300	1,800 ± 275	31.44^†^	0.001^*^
Skin laxity - complete, n (%)	2 (14.3%)	12 (85.7%)	13 (92.9%)	-4.58^‡^	0.001^*^

Post hoc analyses with Bonferroni correction revealed significant reductions in both weight and urine output from the pre‑CNPLT stage to the presurgery and postsurgery stages (all p < 0.001). No statistically significant difference was observed between the presurgery and postsurgery measurements for either parameter (Table 4). Adverse events during the seven-to-eight-day CNPLT phase were mild and infrequent: transient pain/discomfort (resolved with pressure adjustment), superficial skin maceration in folds (managed with barrier cream and redressing), and minor blistering. No severe blistering, necrosis, ulceration, or treatment discontinuation occurred.

**Table 4:** Post hoc analysis with Bonferroni test for outcome parameters ^*^Statistically significant (p < 0.05) CNPLT: circumferential negative pressure lymphedema therapy

Pairwise comparison	Mean difference	t stats	p value
Weight (kg)			
Pre-CNPLT vs. presurgery	18.6	8.12	0.001^*^
Pre-CNPLT vs. postsurgery	22.5	9.84	0.001^*^
Presurgery vs. postsurgery	3.9	1.7	0.106
Urine output (mL/day)			
Pre-CNPLT vs. presurgery	-650	-9.21	0.001^*^
Pre-CNPLT vs. postsurgery	-600	-8.50	0.001^*^
Presurgery vs. postsurgery	50	0.71	0.487

## Discussion

The present study demonstrated the efficacy of CNPLT as a novel, rapid preoperative decongestive strategy for patients with advanced Stage III-IV lymphedema predominantly caused by lymphatic filariasis in a tropical setting. Over a seven-to-eight-day intensive application period, CNPLT achieved statistically significant reductions in limb circumference at all measured sites, with the most pronounced effects at the lateral malleolus and 10 cm above it. This translated into enhanced skin suppleness, with complete laxity resolution increasing from 14.3% to 85.7% preoperatively. Systemic fluid mobilization was evident through a mean body weight decrease of 18.6 kg and elevated urine output, underscoring CNPLT's ability to engage lymphatic pathways physiologically and promote diuresis. These outcomes facilitated safer reductive surgeries, with minor complications, such as wound dehiscence managed conservatively, and no major events, such as necrosis or infection. In a resource-constrained public hospital environment, where prolonged preoperative preparation is often infeasible due to competing acute cases, CNPLT transforms rigid, fibrotic limbs into operable targets within a clinically practical timeframe.

Conventional CDT, the gold standard for lymphedema management, typically yields modest volume reductions over extended periods. For instance, in breast cancer-related lymphedema (BCRL), CDT has been shown to reduce arm volume by a mean of 48 mL in 6.9 months, with combined MLD and exercises [7]. A comprehensive systematic review conducted by Stout et al. [8] assessed empirical evidence regarding CDT in the treatment of lymphedema. The findings of this review indicated that CDT was successful in decreasing limb volume, enhancing quality of life, and reducing complications linked to lymphedema.

In lower extremity lymphedema, similar patterns emerge; pneumatic compression devices as adjuncts to CDT achieve consistent limb volume decreases; however, they often require weeks for noticeable effects [9]. In contrast, CNPLT induced rapid and substantial girth reductions (14-17 cm at mid-calf levels) in just seven to eight days, far surpassing the CDT's timeline and magnitude per unit time, likely due to the continuous subatmospheric pressure (200-250 mmHg) creating mechanical macrostrain on lymphatic structures.

Emerging negative-pressure therapies offer closer parallels to CNPLT. Negative pressure massage therapy in BCRL patients demonstrated superior reductions in lymphedema severity (via L-Dex scores and interlimb volume differences) compared with MLD alone, with pilot data showing large effect sizes [10]. Intermittent pneumatic compression (IPC) has been effective in preventing and treating BCRL, reducing limb volume, and improving joint function, especially at pressures ≤40 mmHg for more than two weeks [11]. Graded negative pressure also facilitates lymphatic flow in lower limb cases, consistent with our observations of enhanced diuresis and tissue softening [12]. However, CNPLT's circumferential, continuous application at higher pressures distinguishes it from intermittent modalities, enabling greater fluid mobilization (evidenced by a weight loss of ~19 kg). Preoperative decongestion strategies emphasize CDT as the first-line treatment before surgeries such as lymphovenous anastomosis (LVA), with combinations such as CDT plus needle electrode stimulation yielding better swelling reduction post-LVA. CNPLT's rapidity addresses CDT's impracticality in high-volume settings, potentially lowering surgical risks such as dehiscence [13,14].

Body mass index (BMI) is a well-established predictor of lymphedema risk, severity, and treatment response, as excess adipose tissue compresses lymphatic vessels, impairs drainage, and promotes fluid retention and fibrosis [15]. In our study cohort with advanced Stage III-IV lymphedema (mean baseline weight 91.5 ± 12.6 kg, suggestive of obesity in most participants), this likely contributed to the pronounced fibrosis, induration, and surgical challenges observed. Remarkably, CNPLT achieved a rapid mean weight reduction of 18.6 kg over seven to eight days, alongside substantial decreases in limb girth and tissue softening, offering a dual benefit by directly addressing both lymphedema volume and obesity-related lymphatic compromise, effects that far exceed the slower, more modest outcomes typically seen with conventional CDT.

Clinically, these findings highlight the importance of routine BMI assessment in lymphedema management: patients should receive tailored precautions, including gradual, sustainable weight control through low-sodium nutrition, lymphatic-safe physical activity (such as supervised walking or aquatic exercises with compression), and avoidance of rapid weight fluctuations to minimize disease progression, reduce cellulitis risk, and optimize long-term surgical and conservative outcomes in high-burden settings such as endemic filariasis regions [16,17].

Clinically, CNPLT holds promise for endemic regions, such as India, where filariasis drives advanced lymphedema. By expediting decongestion, it optimizes surgical candidacy, shortens hospital stays (mean 12-13 days total), and improves postoperative outcomes, including sustained volume control for up to six months. This could reduce the recurrence of cellulitis and enhance functional mobility, alleviating socioeconomic burden in underserved populations. Integration with existing CDT protocols may amplify long-term maintenance.

Limitations include the small exploratory cohort without a randomized control group, which potentially introduces a selection bias. As a feasibility study, effect sizes lack power calculation, and follow-up was limited to six months, precluding long-term durability assessment. Subjective skin assessments and the absence of volumetric imaging may overestimate these benefits. Future multicenter randomized controlled trials comparing CNPLT with CDT or IPC are needed to validate its efficacy and safety.

Implementation of CNPLT in peripheral or low-resource facilities may be limited by a learning curve for proper foam application, seal maintenance, and pressure titration, potentially leading to uneven decongestion or skin injury if staff are inexperienced. Additionally, the need for a reliable continuous negative-pressure device and medical-grade foam introduces initial procurement and maintenance costs that could challenge adoption in primary/secondary care centers in endemic regions, despite being less resource-intensive than prolonged CDT. Rapid fluid mobilization raises the theoretical risk of rebound edema or proximal lymphatic overload if central lymphatic pathways cannot accommodate the sudden load. No clinical rebound was observed in this cohort during the perioperative period or six-month follow-up, possibly due to prompt reductive surgery and continued postoperative compression. Nonetheless, this risk should be monitored in future studies, particularly in patients with long-standing filariasis and suspected central lymphatic dysfunction.

## Conclusions

CNPLT appears to be a promising, rapid preoperative decongestive strategy for advanced Stage III-IV lymphedema, enabling safer reductive surgery with substantial short-term volume reduction and tissue softening in a resource-limited setting. However, it is strictly a preoperative adjunct and not a definitive stand-alone treatment. The use of 200-250 mmHg negative pressure warrants further metabolic and cardiological safety studies, particularly to confirm the absence of fluid overload, electrolyte disturbances, or proximal lymphatic overload before wider adoption or normalization of these pressures. Larger multicenter trials are needed to validate long-term efficacy, safety, and applicability in endemic regions.
